# In Memoriam: Sandy Ford (1950–2015)

**DOI:** 10.3201/eid2204.151336

**Published:** 2016-04

**Authors:** Myron G. Schultz, Alan B. Bloch

**Affiliations:** US Public Health Service and Centers for Disease Control and Prevention (retired), Atlanta, Georgia, USA

**Keywords:** Sandy Ford, HIV/AIDS and other retroviruses, acquired immunodeficiency syndrome, pentamidine, viruses, Centers for Disease Control and Prevention, chemicals and drugs, *Pneumocystis* pneumonia

Sandra Lee Ford (Figure) died April 11, 2015, at 64 years of age. To her children, she was a loving mother; to her co-workers, she was a caring human being and dedicated public health worker; to the world, she was a herald of the AIDS epidemic, playing a major role in alerting the world to the onset of this epidemic.

**Figure:**
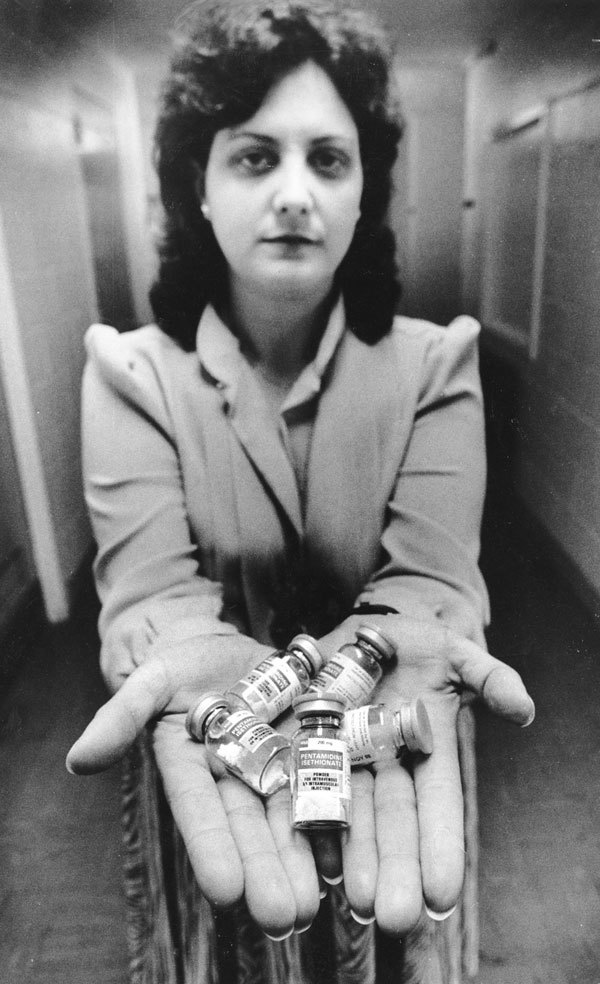
Sandy Ford with vials of pentamidine for distribution to patients with *Pneumocystis* pneumonia.

In January 1979, Sandy took a position in the Parasitic Diseases Division in the Centers for Disease Control, now the Centers for Disease Control and Prevention (CDC). Her job was to respond to requests for drugs that CDC provided through the Parasitic Diseases Drug Service (PDDS). These drugs had been unavailable in the United States. By complying with the Food and Drug Administration’s rigorous requirements for investigational new drugs, CDC was able to import and dispense these drugs and fill a therapeutic gap. One of the 10 drugs in the armamentarium of the PDDS was pentamidine isethionate, which was intended to treat rare, imported cases of African trypanosomiasis. An unanticipated need for pentamidine was its use in treating *Pneumocystis carinii* pneumonia, now known as *Pneumocystis jiroveci* pneumonia or *Pneumocystis* pneumonia (PCP). Pentamidine had replaced sulfa drugs as the treatment of choice for PCP, which had been occurring mainly in infants and children with primary and secondary immune deficiencies and in adults receiving immunosuppressive drugs.

Demand for pentamidine was brisk. Sandy interacted skillfully with hundreds of clinicians across the United States to provide life-saving pentamidine to severely ill patients, and she kept very careful records. She relished the opportunity to help physicians, and she cared deeply about their ill patients. She wanted to be known as a drug technician, a title she coined for herself. On numerous occasions, she said how much she loved her job.

In early 1981, Sandy noticed an increase in the number of requests for pentamidine to treat cases of PCP. The cases were unusual because they were in adult male patients, a departure from the usual cases in children. In one instance, a New York physician asked for a second course of pentamidine to treat a patient with PCP who had not responded to the first dose; these circumstances were unprecedented. Two weeks later, another New York physician told Sandy about 5 male patients who had both PCP and Kaposi sarcoma. Sandy learned that the sexual orientation for all these patients was homosexual. She knew that something unusual was occurring and informed her supervisor of this cluster of cases. In his bestselling book And the Band Played On, which chronicled the discovery of AIDS, Randy Shilts said, “That was how the thorough GS-7 drug technician in Room 161 of the Centers for Disease Control’s Building 6 alerted the federal government to the new epidemic” (*1*). Simultaneously, clinicians in major medical centers were seeing, for the first time, homosexual patients with Kaposi sarcoma, PCP, cytomegalovirus infection, and other opportunistic infections. On June 5, 1981, CDC’s Morbidity and Mortality Weekly Report described 5 severe pneumonia cases observed during October 1980–May 1981 in 3 Los Angeles hospitals (*2*). All 5 patients were young men with PCP, and the sexual orientation of all was homosexual. This publication was historic because it marked the beginning of awareness in the United States of a new, fearsome disease that was eventually given the name of acquired immunodeficiency syndrome (AIDS). The rest, as they say, is history.

Louis Pasteur famously said, “Chance favors the prepared mind.” Chance placed Sandy as a drug technician in CDC’s PDDS at a critical juncture in medical history. Although she had no formal training in medical science, she had a prepared mind and thought like an epidemiologist. When she comprehended that something unusual was occurring, she took appropriate action. In addition to her seminal role in the book And the Band Played On, Sandy was also portrayed in the movie by the same name and was the subject of many magazine and newspaper articles. A heroine of the AIDS epidemic, she continued her career at CDC until retiring in 2008 after 34 years of service. She did not capitalize on her notoriety and conducted her work and her relationships with dignity. She personified the woman in King Solomon’s poem Eshet Chayil, which praises a woman of valor: “A woman of valor, who can find? Her worth is far above jewels…. She is robed in strength and dignity, and cheerfully faces whatever may come…. Wherever people gather, her deeds speak her praise” (Proverbs 31:10–31). 
